# Proteomic analysis of differentially expressed proteins in *Penaeus monodon *hemocytes after *Vibrio harveyi *infection

**DOI:** 10.1186/1477-5956-8-39

**Published:** 2010-07-13

**Authors:** Kunlaya Somboonwiwat, Vorrapon Chaikeeratisak, Hao-Ching Wang, Chu Fang Lo, Anchalee Tassanakajon

**Affiliations:** 1 Center of Excellence for Molecular Biology and Genomics of Shrimp, Department of Biochemistry, Faculty of Science, Chulalongkorn University, Bangkok, Thailand; 2 Institute of Biological Chemistry, Academia Sinica, Taipei, Taiwan, ROC; 3 Institute of Zoology, College of Life Science, National Taiwan University, Taipei, Taiwan, ROC

## Abstract

**Background:**

Viral and bacterial diseases can cause mass mortalities in commercial shrimp aquaculture. In contrast to studies on the antiviral response, the responses of shrimps to bacterial infections by high throughput techniques have been reported only at the transcriptional level and not at the translational level. In this study, a proteomic analysis of shrimp hemocytes to identify differentially expressed proteins in response to a luminous bacterium *Vibrio harveyi *was evaluated for its feasibility and is reported for the first time.

**Results:**

The two-dimensional gel electrophoresis (2-DE) patterns of the hemocyte proteins from the unchallenged and *V. harveyi *challenged shrimp, *Penaeus monodon*, at 24 and 48 h post infection were compared. From this, 27 differentially expressed protein spots, and a further 12 weakly to non-differentially regulated control spots, were selected for further analyses by the LC-ESI-MS/MS. The 21 differentially expressed proteins that could be identified by homologous annotation were comprised of proteins that are directly involved in the host defense responses, such as hemocyanin, prophenoloxidase, serine proteinase-like protein, heat shock protein 90 and alpha-2-macroglobulin, and those involved in signal transduction, such as the14-3-3 protein epsilon and calmodulin. Western blot analysis confirmed the up-regulation of hemocyanin expression upon bacterial infection. The expression of the selected proteins which were the representatives of the down-regulated proteins (the 14-3-3 protein epsilon and alpha-2-macroglobulin) and of the up-regulated proteins (hemocyanin) was further assessed at the transcription level using real-time RT-PCR.

**Conclusions:**

This work suggests the usefulness of a proteomic approach to the study of shrimp immunity and revealed hemocyte proteins whose expression were up regulated upon *V. harveyi *infection such as hemocyanin, arginine kinase and down regulated such as alpha-2-macroglobulin, calmodulin and 14-3-3 protein epsilon. The information is useful for understanding the immune system of shrimp against pathogenic bacteria.

## Background

Shrimps are one of the most economically important species in aquaculture due to their high world-wide demand. With a gradually increasing experience and effort in the development of production technologies, they have become important export products for many countries along the Indo-Pacific coast. The domestic consumption of shrimp has also increased accordingly. Together, these have resulted in a rapid increase in the global shrimp production via aquaculture. Unavoidably, the rise of large scale high density shrimp aquaculture industries has led to several problems in the management of shrimp diseases including pathogens.

The major current viral diseases are white spot syndrome and yellow head diseases, which are caused by white spot syndrome virus (WSSV) and yellow head virus (YHV), respectively [1]. In addition, vibriosis is the major bacterial disease caused by bacteria in the genus *Vibrio *[2]. The outbreaks of these diseases have led to the near or total collapse of the shrimp farming industry throughout the world. Although viral infections typically have more deleterious effects on shrimp farm stocks, vibriosis can also cause mass mortalities of farmed shrimps [3]. In the black tiger shrimp, *Penaeus monodon*, vibriosis caused by *Vibrio harveyi*, a luminous bacterium, usually affects the animal at larval stages. It is considered as an opportunistic pathogen for juvenile and adult shrimps under environmental stress [2,3]. Moreover, the combined infection of pathogenic *Vibrio *spp. and viruses causes a higher and faster mortality rate than viral or bacterial infection alone [4]. Therefore, the elucidation of the shrimp immune responses to vibriosis is of great interest for the prevention and control of infectious diseases in shrimp aquaculture.

Thus far, the response of shrimps upon *Vibrio *infection has been reported only at the transcriptional level [5-7]. Indeed, several groups of *V. harveyi*-responsive genes in shrimps have been identified. They are genes coding for proteins that are involved in diverse cellular functions, for instance, the immune related proteins, metabolic enzymes, structural proteins, proteins involved in signaling and communication, and in apoptosis [6]. Of these, the immune genes are of prime interest. Nevertheless, more information is still needed to gain insight into the defense mechanisms of shrimps against bacterial invasion.

The shrimp hemocytes are the main site where immune defense components are released and the defenses against microbes take place. Besides the secretion of antimicrobial peptides that are considered as the first line of defense against pathogen infections, the other important shrimp defense systems include enzymes and proteins in the prophenoloxidase (proPO) activation and blood coagulation systems. To study in greater depth the immune and related components in the defense system of shrimps, many approaches have been applied. Genomic approaches, such as simple gene cloning, high throughput expressed sequence tag analysis, suppression subtractive hybridization, and others, are important tools for the identification of candidate genes involved in shrimp immunity [8-14]. Nevertheless, the full-genome sequence data of shrimps is seemingly necessary for a more complete search of immune-related proteins.

Recently, a few reports have indicated that proteomic based techniques are a useful alternative method of choice in the identification of shrimp immune-related proteins [11,15-17]. In these studies, differentially expressed proteins from shrimp tissues under hypoxia stress and various pathogenic conditions were examined and compared to those found in shrimps under normal states. Typically, the pathogenicity of viral infections has been the main focus of such studies. For example, the expression profile of proteins from the stomach of WSSV-infected *Litopenaeus vannamei *was determined using 2D-gel electrophoresis (2-DE) and mass spectrometry [16]. Wu et al. [18] used two proteomic approaches, a shotgun 2D-LC-MS/MS and a cleavable isotope-coded affinity tag, to study the differentially expressed cellular proteins from WSSV-infected shrimp epithelium. A 2D-LC-MS/MS approach has also been applied to explore the response of shrimps to WSSV infection in gill tissues [11].

Here, a proteomic analysis of shrimp hemocytes to elucidate the shrimp immune responses at the translational level upon bacterial challenge is reported for the first time. The hemocyte proteins of *P. monodon *whose expression changed upon *V. harveyi *infection were indentified in order to explore the shrimp immune responses against bacterial infection. Then the expression patterns during the course of *V. harveyi *infection of some differentially expressed proteins were further assessed by western analysis and real-time RT-PCR. The results provide important information on shrimp immune responses against bacterial infection.

## Results

### Identification of differentially expressed proteins in hemocytes of *Vibrio harveyi *infected shrimp

A proteomic approach was used in this study to reveal the protein expression in shrimp hemocytes in response to *V. harveyi *infection. The total proteins extracted from the hemocytes of unchallenged and *V. harveyi*-challenged shrimps were separated using 2-DE. In preliminarily work, the appropriate pH range for the first dimension isoelectric focussing (IEF) based separation of the hemocyte proteins was determined using a 13-cm IPG strip of pH 3 - 10. Since most of the protein spots were present in the pH range of 4 - 7 (data not shown), subsequent 2-DE resolutions utilised 13 cm IPG strips of pH 4 - 7 for better separation and resolution. The amount of protein and staining procedure used in the experiment were also empirically optimised. It was found that 200 and 700 μg protein samples were required for the optimal SYPRO Ruby and colloidal Coomassie Blue G-250 staining, respectively. Consequently, we separated 200 μg protein samples and stained the gels with SYPRO Ruby.

**Figure 1:**
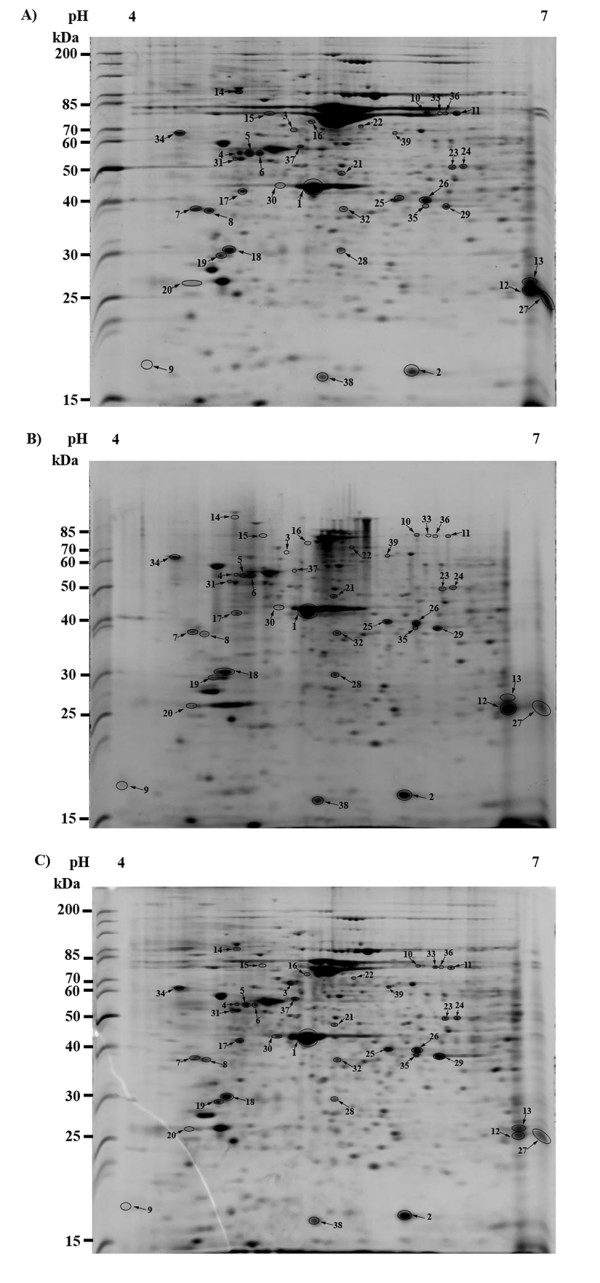
**Representative 2-DE resolution of the protein spots of *Penaeus monodon *hemocytes with or without *Vibrio harveyi *challenge at 24 and 48 hpi**. The hemocyte protein expression profile of (A) unchallenged shrimps was compared to that of *V. harveyi*-challenged shrimps at (B) 24 hpi and (C) 48 hpi. The 27 differentially expressed protein spots and a further 12 control spots selected are circled and numbered (see Table 1).

Under the appropriately set conditions, 200 μg of hemocyte protein samples, each from three individuals of unchallenged or *V. harveyi*-challenged shrimps at 24 or 48 h, were separated through 2-DE. Three gel replicates, representing nine individual shrimps, were analyzed at each time point. The gel images were compared and analyzed. The patterns of protein expression in unchallenged and *V. harveyi*-challenged shrimp hemocytes appeared largely similar but some clear differences in protein expression levels of certain protein spots were evident (Figure 1). These were grouped, in terms of their expression level relative to that in the control shrimps as significantly up-regulated, significantly down-regulated and 'constant' protein spots, as outlined in the methods. Thirty-nine protein spots were excised for further analysis by the nano-LC-ESI-MS/MS (Figure 1). Of these, 10 spots were up-regulated, 17 were down-regulated and 12 ranged from weakly up-regulated or down-regulated (10) to no detectable difference (2) and were selected as the control constantly expressed protein spots (Figure 1). Nevertheless, 10 of these protein spots were unable to be identified to annotated proteins, leaving just nine, twelve and eight annotated up-regulated, down-regulated and constant proteins, respectively (Table 1).

**Table 1:** Thirty nine selected 2-DE spots (proteins) from the hemocytes of *Vibrio harveyi*-challenged *Penaeus monodon*, resolved by 2-DE and identified by LC-nano ESI-MS/MS.

**Spot no**.	**Predicted MW (Da)**	**Predicted pI**	Protein hit	Mowse Score/no. of match peptides	**Accession no**.	Fold change*	
						
						24 h	48 h
**Up-regulated protein spots**							
33	78700	6.05	prophenoloxidase 2 [*Litopenaeus vannamei*]	71/2	ABQ45957	U	U
30	41822	5.11	actin 2 [*Penaeus monodon*]	240/7	AAC78682	U	U
3	74934	5.27	hemocyanin [*Litopenaeus vannamei*]	99/4	CAA57880	-1.35	+6.88
16			unknown			-2.92	+4.11
35	40087	6.05	arginine kinase [*Penaeus monodon*]	485/13	AAO15713	+1.85	+4.09
29	40087	6.05	arginine kinase [*Penaeus monodon*]	704/32	AAO15713	+2.33	+3.16
2	17142	6.74	twinstar [*Drosophila melanogaster*]	150/8	NP_477034	+1.88	+1.20
37	49283	4.92	tubulin alpha chain [*Oncorhynchus keta*]	94/3	P30436	-1.38	+1.88
4	52810	5.08	serine proteinase-like protein [*Penaeus monodon*]	119/5	ABD62888	+1.69	-1.25
32	37134	6.73	transaldolase [*Bombyx mori*]	110/1	NP_001040544	+1.60	+1.58
**Down-regulated protein spots**							
20	32431	5.04	alpha-2-macroglobulin [*Penaeus monodon*]	59/1	AAX24130	D	D
9	16671	4.04	calmodulin [*Patinopecten *sp.]	54/3	0711223A	D	-13.89
19	29054	4.65	14-3-3 protein epsilon [*Danio rerio*]	114/5	NP_997770	D	+1.12
12			unknown			-1.08	-3.75
14	96846	4.73	karyopherin (importin) beta [*Nematostella vectensis*]	109/3	XP_001636221	-8.14	-3.21
8			unknown			-3.42	-2.70
36	78700	6.05	prophenoloxidase 2 [*Litopenaeus vannamei*]	119/3	ABQ45957	-3.49	-2.64
11	78700	6.05	prophenoloxidase 2 [*Litopenaeus vannamei*]	127/3	ABQ45957	-2.31	-2.87
7			unknown			-1.59	-2.73
13	24283	6.52	GTP-binding nuclear protein Ran (GTPase Ran) (Ras-like protein TC4) [*Brugia malayi*]	104/2	P38542	-1.52	-2.75
27			Unknown			-1.35	-2.39
6	52810	5.08	serine proteinase-like protein [*Penaeus monodon*]	84/2	ABD62888	-1.37	-2.42
10	78426	5.83	Prophenoloxidase-1 [*Penaeus monodon*]	973/20	AAM77689	-1.88	-2.32
31	56040	5.10	ATP synthase subunit beta, mitochondrial precursor [*Hemicentrotus pulcherrimus*]	411/7	Q25117	-2.06	-1.17
5	52810	5.08	serine proteinase-like protein [*Penaeus monodon*]	119/5	ABD62888	-1.92	-1.48
15	83278	4.89	heat shock protein 90 [*Metapenaeus ensis*]	484/17	ABR66910	-1.17	-1.59
25			Unknown			-1.40	-1.62
**Constant protein spots**							
38	28203	5.89	thymosin beta-11 [*Penaeus monodon*]	419/10	BI018085	+1.41	+0.98
34	46851	4.41	calreticulin [*Gallus gallus*]	82/3	AAS49610	-1.17	+1.48
28	31358	5.42	cytosolic manganese superoxide dismutase [*Penaeus monodon*]	421/12	AAW50395	+1.18	-1.50
26	40087	6.05	arginine kinase [*Penaeus monodon*]	103/3	AAO15713	+1.03	+1.16
24	47235	6.18	phosphopyruvatehydratase [*Penaeus monodon*]	452/9	AAC78141	+1.06	+1.01
17	32574	4.78	hypothetical protein LOC550536 [*Danio rerio*]	58/3	NP_001017838	+1.24	+1.12
18	32420	4.70	tropomyosin [*Locusta migratoria*]	305/17	P31816	+1.47	+1.06
1	41841	5.30	beta-actin [*Litopenaeus vannamei*]	1083/80	AAG16253	+1.46	+1.32
39			Unknown			+1.12	+1.21
21			Unknown			-1.07	-1.36
22			Unknown			+1.21	+1.14
23			Unknown			-1.24	+1.19

* indicates the decrease in the spot intensity at a given time point after *V. harveyi *infection compared to that of normal shrimp.

+ indicates the increase in the spot intensity at a given time point after *V. harveyi *infection compared to that of normal shrimp.

U indicates the up-regulated protein spot detected only in the protein extracts of infected animals.

D indicates the down-regulated protein spot detected only in the protein extracts of normal animals.

### Differentially expressed hemocyte proteins after *V. harveyi *infection

The proteins showing significant changes in their expression levels upon *V. harveyi *infection are summarized in Table 1. The 21 identified proteins that varied in expression levels showed diverse annotated functions, including the immune related proteins (proPO-1, proPO-2, serine proteinase-like protein and hemocyanin), stress response protein (heat shock protein 90), serine proteinase inhibitor (alpha-2-macroglobulin), cytoskeletal proteins (actin, tubulin and twinstar), enzymes involved in energy (argenine kinase (AK)) and carbohydrate (tal1) metabolism, and proteins involved in nuclear cytoplasmic transport (GTPase Ran), and mediators of signal transduction (14-3-3 protein epsilon and calmodulin) or intracellular trafficking and secretion (karyopherin beta).

Among the proteins identified, the up-regulated proteins, the more acidic proPO-2 spot (spot 33) and actin 2 were found to be expressed only at 24 and 48 h post *V. harveyi *injection (hpi) but not in the unchallenged shrimps. Moreover, the translational level of hemocyanin in the hemocytes of *P. monodon *was dramatically increased by 6.88-fold at 48 hpi after an initial slight down-regulation (-1.35-fold) at 24 hpi. In addition, both the up-regulated AK spots revealed a late response, being strongly up-regulated at 48 hpi, (spots 29, 35).

For the down-regulated protein spots, alpha-2-macroglobulin was found to be down-regulated at 24 and 48 hpi such that it could only be detected in the unchallenged shrimps, whilst calmodulin and 14-3-3 protein epsilon were more transiently down-regulated in that they were not observed at 24 hpi but then expressed at 48 hpi at very low (calmodulin) or normal (14-3-3-epsilon) levels (Table 1). Finally, the importin homologue (spot 14) showed a strong earlier down-regulation, with a marked down-regulation at 24 hpi and then recovering partially by 48 hpi. The down-regulation of the two proPO-2 isoforms (spots) is discussed below.

### Western blot analysis and validation of the protein expression levels

The proteomic data were preliminary validated by determining the protein expression level of hemocyanin by quantitative western blots. This protein was selected, along with the 'constant spot' protein of phosphopyruvate hydratase as a reference control, on the basis that in each case a specific antibody was available.

**Figure 2:**
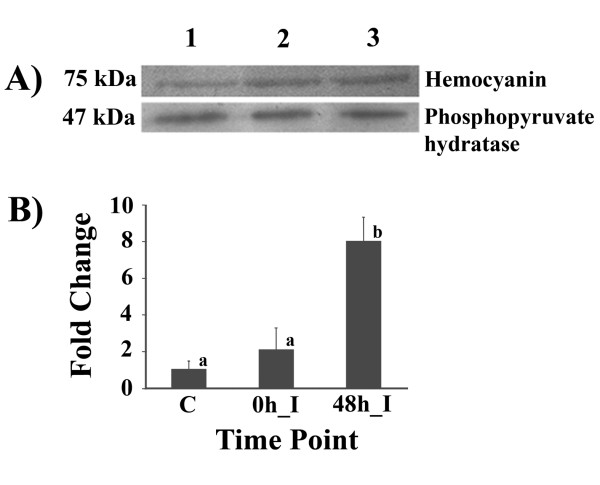
**Western blot analysis of hemocyanin protein expression levels in hemocytes of *Vibrio harveyi*-challenged *Penaeus monodon***. (A) The expression of hemocyanin protein in the hemocytes of control (saline-injected) and *V. harveyi*-challenged shrimps at 0 and 48 hpi, as assayed by western blot analysis using an antibody specific to hemocyanin. The protein expression level at each time point was then normalized to that of phosphopyruvate hydratase. (B) Fold change of hemocyanin expression level after *V. harveyi *infection at 0 (0 h_I) and 48 h (48 h_I) compared to that of control shrimp (0 h_N) was shown. The data is shown as the mean ± 1S.D. and is derived from 3 repeats. Means with different letters are significantly different (*p *< 0.05).

The results, summarised in Figure 2, show a remarkable eight-fold increase in hemocyanin protein levels at 48 h post *V. harveyi *infection compared to that at 0 hpi. The western blot result seems to agree well with the proteomic results, where the expression of hemocyanin protein was increased by 6.88 fold (Table 1). Although this represents only a single verification, and is based on the somewhat reasonable assumption that phosphopyruvate hydratase protein levels do not change, the proteomic data is nevertheless deemed to be likely to be more or less acceptable.

### Real-time RT-PCR of the differentially expressed protein genes

Besides expression at the post translational level, we determined the expression of certain genes of interest at the transcriptional level to see if they were related. The 14-3-3 protein epsilon and alpha-2-macroglobulin were interesting because their protein levels decreased significantly at 24 h after *V. harveyi *injection, and hemocyanin whose protein expression levels increased at 48 hpi, were chosen for the quantitative real-time RT-PCR analysis to evaluate their transcript expression. The temporal gene expression analysis was examined in *V. harveyi*-challenged *P. monodon *at various time points (0, 6, 24 and 48 hpi) and compared to that in the saline injected control shrimps. The β-actin gene was used as an internal control.

**Figure 3:**
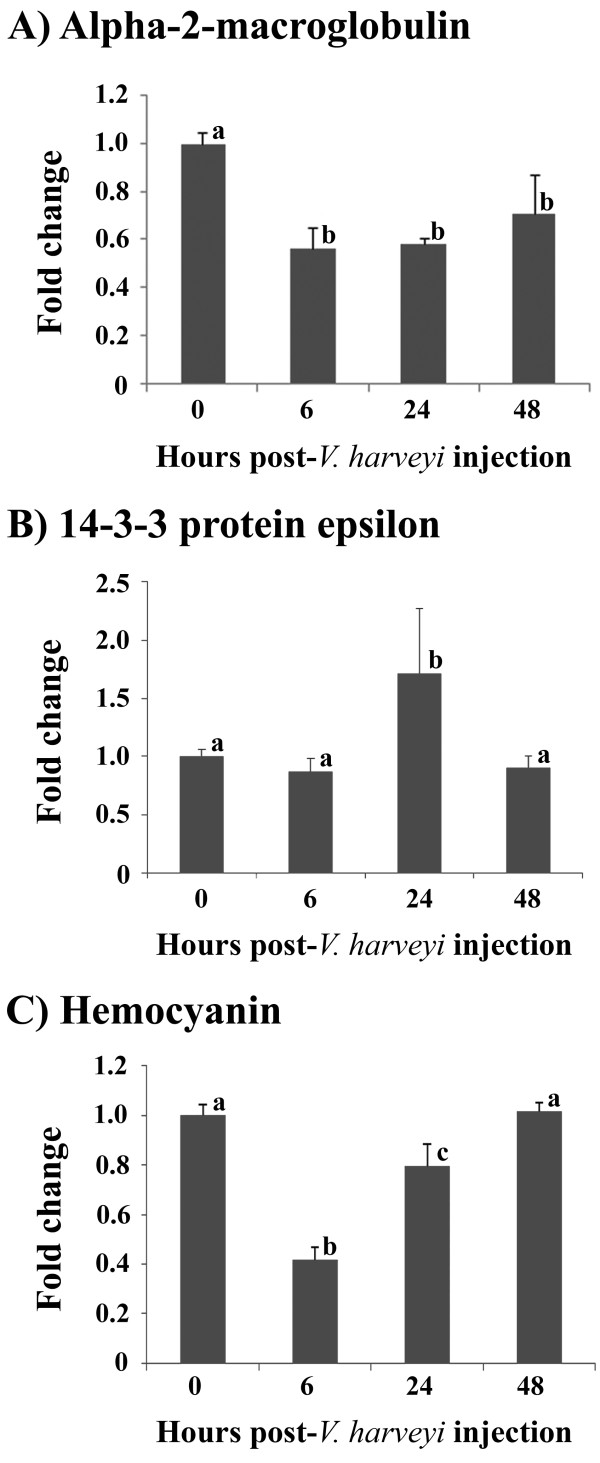
**Time course analysis of alpha-2-macroglobulin, the 14-3-3 protein epsilon and hemocyanin gene transcript levels after *Vibrio harveyi *challenge, assayed using quantitative real-time RT-PCR**. Total RNA was extracted from hemocytes of saline-injected and *V. harveyi*-challenged shrimps collected at 0, 6, 24 and 48 hpi and the cDNA was then synthesized. The mRNA expression levels of (A) alpha-2-macroglobulin, (B) 14-3-3 protein epsilon and (C) hemocyanin upon *V. harveyi *challenge were determined using the beta-actin gene as an internal control. Data are shown as the mean ± 1S.D. Means with different letters are significantly different (*p *< 0.05).

The alpha-2-macroglobulin transcription decreased significantly at 6 hpi to a 0.57-fold lower than that at 0 hpi and remained at this level at 24 hpi with a slight numerical but not statistically significant increase at 48 hpi to 0.71-fold lower than at 0 hpi, as illustrated in Figure 3A. The 14-3-3 protein epsilon transcription expression levels were significantly up-regulated, 1.72-fold over that of the control, by 24 hpi (Figure 3B). The hemocyanin transcript levels decreased significantly at 6 hpi to a 0.42 fold lower than that at 0 hpi and then returned to normal level at 48 hpi (Figure 3C). It was found that the levels of transcription of the alpha-2-macroglobulin gene were found to relate well with that of its translation products in that it was down-regulated upon *V. harveyi *infection, whilst the levels of hemocyanin and 14-3-3 protein epsilon transcripts were not corresponded with that of their translation products.

## Discussion

With advancing analytical techniques, proteomic analysis has become a new frontier of study in molecular biology in which the differences or changes in protein expression patterns of organs, cells or subcellular compartments can be readily elucidated to a high to a reasonable level of coverage. Under several circumstances, such studies can provide an understanding of the response of cells to various external factors. The analysis, generally, involves 2-DE in conjunction with mass spectrometry for protein separation and protein identification, respectively [19].

In this study, we used a proteomic approach to analyze the complex proteome of shrimp hemocytes to evaluate those proteins whose expression was significantly up-regulated or down-regulated after systemic challenge with the bacterial pathogen *V. harveyi*. Among these, hemocyanin (~75 kDa) which is the most abundant protein in the hemolymph, was found to be one of the prominent proteins, in accord with a previous report [20], even though an extensive washing of the hemocytes was performed to remove hemolymph prior to protein extraction and preparation. A total of 27 differentially expressed protein spots, 10 up-regulated and 17 down-regulated, plus 12 other spots that ranged from unchanged to only weakly altered expression levels as controls were selected and processed via mass spectrometry for annotation. From all 39 selected samples, however, 10 were not able to be identified from the annotated databases, leaving 9, 12 and 8 up-, down- and non-regulated protein spots, respectively, as annotated. Since the genome sequence of the shrimp is still unavailable, the origins of these 10 unidentified protein spots are uncertain although most of them are probably shrimp proteins. It is of note that several *Vibrio *sp. genomes, including *V. harveyi *and some of its phages, are available allowing, subject to equivocal caveats of their correct annotation and conserved nature across isolates, exclusion of their proteins and some of their phage encoded proteins too. Moreover, nine of the protein spots were annotated to just three proteins, that being three isoforms that differ in pI and or mass of each of proPO-2, AK and serine protease-like protein (Table 1). Assuming the more likely correct annotation of these different protein spots, rather than miss annotation of related proteins or conserved domains, these differences then probably arise from posttranslational modifications of a portion of the same protein population, and this is discussed below.

From the protein profiles obtained, those with altered protein expression levels (or posttranslational modifications) are expected to be involved directly or indirectly in shrimp immune responses. These proteins participate in various cellular functions, including immunity such as hemocyanin, prophenoloxidase, serine proteinase-like protein, heat shock protein 90 and alpha-2-macroglobulin as well as the cytoskeletal proteins including actin 2, twinstar and tubulin alpha chain. Among the immune proteins, those involved in melanization and phagocytosis were prominent suggesting the importance of these immune processes in antibacterial defence. In the previous studies, melanization of bacteria was shown to be a critical defense reaction in invertebrates and appears to be associated with phagocytosis [21,22].

The proPO activating system is an important immune response that produces melanin and reactive oxygen species to kill, trap and eliminate invading microorganisms. For invertebrates, this system is of the utmost importance in the immune response against bacterial infections. Two different proPOs, proPO-1 and proPO-2, which each play crucial roles in the proPO activating system, have been identified in several shrimp species including *P. monodon*, *Fenneropenaeus chinensis *and *L. vannamei *[23-25].

In *P. monodon*, the proPOs were found to be essential against *V. harveyi *infection by gene knockdown experiments [24]. In this study, we found that the protein spots identified as proPO-1 and proPO-2 (1 and 3 spots, respectively, out of 39 protein spots) were the most dramatically differentially expressed immune related proteins upon *V. harveyi *infection. The three spots of proPO-2 (spot nos. 33, 36 and 11) were the same protein judging from their partial amino acid sequences and molecular masses, but presumably with different posttranslational modifications. proPO-2 (spot no. 33) was up-regulated from no detectable expression in control (uninfected) hemocyctes, while the two other proPO-2 isoforms (spot nos. 11 and 36) were down-regulated, suggesting a possible pI altering posttranslational modification of the proPO-2 of spot 11 to 36 and 33.

In *L. vannamei*, the expression of the proPO-I gene in the hemocytes of *Vibrio alginolyticus *infected shrimps was found to be up-regulated at an early phase of infection (6 and 12 hpi), whereas that of proPO-II was reduced significantly at 3 hpi and subsequently increased at 12 hpi, and by 24 hpi, both proPO-I and proPO-II showed no difference in the expression as compared to the control shrimp [26]. This more or less corresponds to our observation where at the late phase of bacterial infection (24 and 48 hpi) the two spots of proPO-2 (spot nos. 11 and 36) and that of proPO-1 (spot no. 10) were found to have a low protein expression level. Considering the expression of the up-regulated form of the proPO-2 (spot no. 33) observed at the late phase of *Vibrio *infection, we speculated that it was the inactive form of proPO-2 that the shrimp produced to restore the level of proPO-2 in the hemocyte in order for the shrimp to promptly fight against another invasion, if any.

The serine proteinase-like protein (spot nos. 4, 5 and 6) was observed as differentially expressed protein spots. They were down-regulated as the infection progressed, albeit with a somewhat up-regulated expression level for spot no. 4 at 24 hpi. They were found to be identical proteins from the partial peptide sequences, and if so likely represent different pI changing posttranslational modifications. Their amino acid sequences matched the serine proteinase homolog 516 (SPH516) from *P. monodon*, which could interact with a putative metal ion-binding domain of the yellow head virus [27]. The SPH516 protein is nearly identical in sequence to the masquerade-like serine proteinase homolog, *Pm*MasSPH. Amparyup et al. [28] reported that the *Pm*MasSPH transcript in the hemocyte of *P. monodon *was up-regulated 24 h after *V. harveyi *injection. Therefore, spot no. 4, which showed corresponding expression levels at both the transcription and translation levels, may be the active form of this serine proteinase homolog. Recently, it was shown that the *Pm*MasSPH protein was a multifunctional immune effector. The C-terminal SP-like domain of *Pm*MasSPH possesses a hemocyte adhesion activity and binding activity to *V. harveyi *and lipopolysaccharide, the bacterial cell wall component. The N-terminal region exhibits an anti-Gram-positive bacteria activity. Moreover, the *Pm*MasSPH protein also displays an opsonic activity upon bacterial clearance from the shrimp circulation [29]. Taken together these results support the likely role if not importance of SPH in the shrimp immune response to bacterial infection.

The other two up-regulated protein spots, nos. 29 and 35, were identified as AK, an enzyme important in cellular energy metabolism in invertebrates. The most significant up-regulation was found for AK spot no. 35. These three protein spots had identical peptide fragment sequences. However, this later constantly expressed AK spot 26 was of a slightly higher MW than AK spots 29 and 35, which differ from each other in pI. Thus, these may represent separate alleles or isoforms under separate regulation and not simple interchanges of posttranslational modifications. Previously, the synthesis of AK in the stomach of *P. monodon *was shown not to be affected by WSSV infection at 48 h [16]. In contrast, AK up-regulation was observed in the gills of YHV-infected *P. monodon *[30]. In addition, AK protein levels in the plasma of *F. chinensis *exhibited the most significant changes, as assayed by 2-DE, at 45 min and 3 h after laminarin stimulation [17]. The up-regulation of AK protein levels likely indicates that energy was required in response to infection.

Alpha-2-macroglobulin (spot no.20) was detected only in unchallenged shrimp indicating that the protein was down-regulated upon bacterial challenge. Alpha-2-macroglobulin is a family of protease inhibitors that participates in innate immune system as a regulator of the proPO system [31]. Also it acts against invading parasites by inactivating and clearing the protease virulence factors of parasites. After trapping the target protease, alpha-2-macroglobulin and its bound protease are degraded in secondary lysosomes [32]. In *P. monodon*, alpha-2-macroglobulin has been also shown to have the ability to bind to Pm-syntenin whose transcript was up-regulated upon WSSV infection [33]. According to our result, alpha-2-macroglobulin protein was expressed only in the unchallenged shrimp while its transcript was still detectable even at lower level upon *V. harveyi *infection. We speculated that alpha-2-macroglobulin and its bound protease might be in the degradation process during 24 and 48 hpi which caused the disappearance of the protein on the 2DE-gel.

Heat shock proteins (HSPs) are ubiquitous and highly conserved among various organisms. They play roles in the stress response in animals by acting as molecular chaperones which help refolding the misfolded proteins. HSP 90, is a family of HSPs in which its family members are either constitutive or inducible genes. Apart from being an important cytoplasmic chaperone, HSP 90 is essential for many cellular processes such as cell proliferation, differentiation, and apoptosis [34,35]. HSP 90 has been currently identified in *P. monodon *and the expression analysis revealed that it was heat inducible gene [36]. Upon heat-killed *V. harveyi *challenge, the HSP 90 transcript level in gill was obviously induced as compared to those of saline-injected shrimp at 3, 12 and 24 hpi and appeared to be expressed at the normal level at 72 hpi [37]. However, the data on expression of HSP 90 in hemocyte of bacterial challenge shrimp is not available. Herein, proteomic data indicated that the level of expression of HSP 90 protein (spot no.15) in shrimp hemocyte after *V. harveyi *challenge was unchanged at 24 hpi but slightly decrease at 48 hpi.

Cytoskeletal proteins, including actin 2 (spot no. 30), twinstar (actin depolymerizing factor (ADF)/cofilin-like) (spot no. 2) and tubulin alpha chain (spot no. 37), were found to be up-regulated significantly upon *V. harveyi *infection, especially that for actin 2 that was up-regulated from no detectable expression in the control shrimp hemocyctes. It is well-known that actin polymerization is required for phagocytic process [38], which are one of the important innate immune reactions in all multicellular organisms including shrimps. Indeed, it has been shown that semi-granulocytes and granulocytes are responsible for the phagocytosis of invading *V. alginolyticus *in the shrimp *Penaeus indicus *[39], and that this requires remodelling of the actin skeleton. Meanwhile, the actin dynamics are regulated by a complex mechanism through the actions of several actin-binding proteins. The ADF/cofilin is one of the essential actin-binding proteins that participate in actin filament assembly by enhancing the depolymerization of actin monomers and so accelerating the filament treadmilling [40]. The significant increase in the production of actin2 and ADF/cofilin at 24 hpi that was observed with *V. harveyi *infection thus likely reflects the induction of phagocytosis in shrimp hemocytes in response to infection with *V. harveyi*.

The Ran GTPases are small G proteins that function to regulate nucleocytoplasmic transportation [41]. In shrimps, a Ran GTPase gene of *Penaeus japonicus *(PjRan) was reported to be up-regulated in WSSV resistant and WSSV-infected shrimps, implying its potential involvement in the shrimp antiviral immune response [42]. Very recently, it was found that Ran GTPase might help shrimps to combat viral infection via regulation of the hemocytic phagocytosis by interacting with an actin-associated molecular motor, the myosin light chain [43]. In contrast to the viral response, the expression of Ran GTPase protein (spot no. 13) upon *V. harveyi *challenge was noticed in this study to be reduced as the course of infection progressed. The reason why Ran GTPase protein expression was decreased in shrimp hemocytes at the late phase of bacterial infection remains unclear and in need of further investigation.

## Conclusions

A proteomic analysis of shrimp hemocytes revealed several proteins whose expression levels were altered in response to *V. harveyi *invasion. These proteins were likely to be involved in various cellular functions, including immune functions, such as proPO-1, proPO-2, serine proteinase-like protein, hemocyanin, heat shock protein 90 and alpha-2 macroglobulin. Interestingly, among the immune-related proteins, those involved in the proPO activating system and phagocytosis were dominant, implying a major role of these innate immune reactions in the antibacterial defense. The information provided here enhances our knowledge on the molecular responses of shrimps against pathogenic bacteria that will lead to a better understanding of the pathogenesis of vibriosis.

## Methods

### Shrimp and bacterial infection

For proteomic analysis and real-time RT-PCR, sub-adult *P. monodon *(approximately 3-months old, 15 - 20 g of live (wet) body weight) were obtained from the Thailand National Shrimp Improvement and Breeding Center, National Center for Genetic Engineering and Biotechnology (BIOTEC) at Chaiya County, Surathani Province, and from the Shrimp Quarantine and Broodstock and Larval Development Research Center at Walailuk University, Nakornsrithammarat Province, Thailand, respectively. They were acclimatized in aquaria at ambient temperature (28 ± 1°C) in air-pumped circulated artificial seawater with a salinity of 15 ppt for at least 3 days before experimental use.

The shrimp pathogenic *V. harveyi *strain 639 was prepared as described by Ponprateep et al. [44]. Microbial challenge was carried out by intramuscular injection into the fourth abdominal segment of individual shrimps with a lethal dose (86.6% mortality within seven days) of 100 μl of live *V. harveyi *639 (10^6 ^CFU) diluted in sterile saline solution (0.85% (w/v) NaCl). To confirm the presence of luminous bacteria in the *V. harveyi*-challenged shrimps but not in the unchallenged shrimp, the hepatopancras lysate of each shrimp was streaked onto the tryptic soy broth agar plate (the selective media). After incubation at 30°C for overnight, the plates were observed for the luminous bacteria in the dark.

For the real-time RT-PCR and Western blot analysis, control shrimps were injected with bacteria-free normal saline solution but otherwise treated the same as the *V. harveyi *challenged shrimps.

### Protein sample preparation

Hemolymph was collected from nine individual shrimps for each experimental group, either unchallenged or 24 and 48 h post *V. harveyi *challenge (hpi), using an equal volume of MAS solution (27 mM sodium citrate, 336 mM NaCl, 115 mM glucose and 9 mM EDTA, pH 7.0). Hemocyte was collected, extensively washed twice with MAS solution and then resuspended in lysis buffer (8 M urea, 2 M thiourea, 0.2% (v/v) Triton X-100 and 50 mM DTT supplemented with 1 × proteinase inhibitor mix (GE healthcare)). After that the supernatant was collected by centrifugation at 12,000 × g at 4°C for 20 min and precipitated with a 3:1 (v/v) ice-cold acetone/methanol mixture. The pellet was washed with cold acetone, and resuspended in sample buffer (8 M urea, 2 M thiourea, 4% (w/v) CHAPS supplemented with 1× proteinase inhibitor mix). The soluble protein fractions from three individuals were pooled and kept at -80°C. Protein concentration was determined using a 2-D Quant Kit (GE healthcare).

### Two-dimensional gel electrophoresis (2-DE)

A total of 200 μg of protein extract was mixed with rehydration buffer (8 M urea, 2 M thiourea, 4% (w/v) CHAPS and 1% (w/v) bromophenol blue). Three IPG strips (nonlinear pH 4 - 7; 13 cm long) (GE healthcare) were used for each experimental group representing nine individuals per group. They were run simultaneously on an Ettan IPGphor II IEF system (GE healthcare). The strips were actively rehydrated for 16 h at 20°C and the IEF was continued using the following step voltage focusing protocol: 2 h each at 300 V, 500 V, 1000 V and 4000 V followed by 10 h at 8000 V.

The focused IPG strips were sequentially equilibrated in a SDS equilibration buffer containing 10 mg/ml DTT and 2.5 mg/ml iodoacetamide for 15 min each. Then, they were placed onto an SDS-PAGE (4% (w/v) acrylamide stacking gel, pH 6.8, and 12.5% (w/v) acrylamide separating gel, pH 8.8). After the second dimensional separation, the gels were fixed and stained with SyproRuby (Invitrogen) and then scanned using a Typhoon 9400 scanner (GE healthcare). The gel images were then analyzed using ImageMaster 2D Platinum software (GE healthcare). The spots were detected, matched across the gels and re-checked and edited manually to ensure the correctness. The percent volume (% vol), as the normalized value of the intensity volume of each spot to the total intensity volume of all spots detected on the gel, was statistically analyzed via Student's t test (p < 0.05). To identify the differentially expressed protein spots, spot intensities of infected groups were compared to those of normal groups and divided into three groups, in terms of their expression level relative to that in the control shrimps as; significantly up-regulated, significantly down-regulated and the weakly but not significantly or not detectably 'constant' protein spots. For this classification, the criterion for an up-regulated protein spot was that the % vol of the spot from the challenged shrimps was at least1.5-fold higher than that of the unchallenged shrimps. For down-regulated protein spots; the % vol of the spot from the challenged shrimps to that of the unchallenged shrimps must be at least 1.5-fold lower. Spots that did not fall into either of these two categories, that is were only weakly changed in either direction or showed no change in expression level at all, were considered as the constantly expressed proteins.

### Protein annotation

The differentially expressed protein spots were excised from the gel and subjected to in-gel trypsin digestion, as described by Wang et al. [16]. The tryptic peptides were analyzed by a nano-LC-ESI-MS/MS. The data obtained were searched against the NCBI protein and EST sequence databases using the MS/MS ion search tool of the MASCOT program. The parameters used for the MS/MS ion search were a peptide mass tolerance of 0.25 Da and up to one missed cleavage allowed. Methionine oxidation and cysteine carbamidomethylation were chosen as the variable modification parameters. The protein was annotated if significant hits, as defined by the Mascot probability analysis, were obtained. Moreover, the search results were always checked for confirmation with the predicted potential target protein's MW/p*I *values against those of the corresponding identified spot.

### Quantitative real-time RT-PCR

Total RNA was extracted from the hemocytes of *V. harveyi*- and saline-injected control shrimps at 0, 6, 24 and 48 h post infection (hpi), as described by Ponprateep et al. [44]. At each time point, equal amounts of total RNA from three individual shrimps were pooled. Then, 1 μg of the pooled total RNA was reverse transcribed using RevertAID™ first strand cDNA synthesis kit (Fermentas).

Quantitative real-time RT-PCR was performed on an iCyclerQ™ Real-Time Detection System (Bio-Rad Laboratories). Target genes, shown in Table 2, were amplified in triplicate in the reaction mixture containing 1 × Maxima™ SYBR Green qPCR Master Mix (Fermentas), an appropriate concentration of the specific primer pairs and optimised PCR amplification conditions, as noted in Table 2. The expression level of the target transcript was normalized to that of the internal control, the β-actin gene, and the relative expression ratio was determined according to Pfaffl [45]. Statistical analysis was performed using ANOVA and Post Hoc test with a *p *value < 0.05 being accepted as significant.

**Table 2:** List of primers and optimal PCR amplification conditions used for the quantitative Real-time RT-PCR

Gene	Primer name (Sequence (5'-3'))	Final concentration (nM)	Temperature (°C)/time (sec)		
			
			Denaturation	Annealing	Elongation
Alpha-2-macroglobulin	A2M_F (CCTCATATCCGGCTTCATCC)	100	95/10	60/20	72/10
	A2M_R (CCGTGAACTCCTCGATGTAG)	100			

14-3-3 protein epsilon	14-3-3_F (GTATCTTGCCGAGACCGCCACT)	200	95/10	63/15	72/20
	14-3-3_R (CAATGTCGCTGGCTGCCTTGT)	200			

Hemocyanin	Hemocyanin_F [46] (AAGTGCTCGGAATCTTCGGTAA)	200	95/10	60/15	72/10
	Hemocyanin_R [46] (CCTGCCTCGATCTTTGCAA)	200			

Beta actin	Beta_F (GAACCTCTCGTTGCCGATGGTG)	200	95/30	60/30	72/45
	Beta_R (GAAGCTGTGCTACGTGGCTCTG)	200			

### Western blot analysis

Five or 15 μg, for hemocyanin and, as an internal control, phosphopyruvate hydratase, respectively, of the pooled hemocyte protein from 20 individual unchallenged or *V. harveyi*-challenged shrimps at 0 and 48 hpi were separated on a 10% (w/v) acrylamide SDS-PAGE gel and then transferred onto nitrocellulose membranes. Hemocyanin and phosphopyruvate hydratase were each detected using a specific rabbit polyclonal antibody at a dilution of 1:10,000 for 1 h at 37°C and at 1:20,000 for 3 h at 65°C, respectively. These conditions were empirically optimised in the laboratory (data not shown). The secondary antibody was AP-conjugated goat anti-rabbit IgG (Jackson Immuno Research Laboratories). The positive band was detected by adding Lumi-Phos™ WB substrate (Pierce) and exposing the membrane to the X-ray film. The band intensity was quantified by ImageQuant™ TL (GE healthcare). The experiment was done in triplicate. The band intensity of the hemocyanin protein expressed at each time point was normalized to that of the internal control. Statistical analysis was performed by ANOVA and Post Hoc test, with a *p *value < 0.05 being accepted as significant.

## Competing interests

The authors declared that they have no competing interests.

## Authors' contributions

KS contributed to the experimental design, performed the experiment, data analysis and manuscript preparation. VC participated in performing the experiment, data analysis and helped to draft the manuscript. HCW provided technical assistance in the experimental part, help in data interpretation and drafting the manuscript. CFL took part in supervision, supported the cost of peptide analysis by mass spectrometry, provided the antibodies for all tests and drafted the manuscript. AT was responsible for experimental design, supervision and preparation of the manuscript. All authors read and approved the final manuscript.
